# QT interval prolongation and its related factors before and after receiving lopinavir-ritonavir in the COVID-19 era: a historical cohort study

**DOI:** 10.1016/j.virusres.2026.199739

**Published:** 2026-04-29

**Authors:** Ali Jangjou, Payman Izadpanah, Mostafa Moqhadas, Hosein Faramarzi, Mohammad Hossein Fahimi, Hamid Ghazipoor, Hadid Hamrah, Hesam Kamyab

**Affiliations:** a Department of Emergency Medicine, School of Medicine, Namazi Teaching Hospital, Shiraz University of Medical Sciences, Shiraz, Iran; b Department of Cardiology, Shiraz University of Medical Sciences, Shiraz, Iran; c Department of Community Medicine, School of Medicine, Shiraz University of Medical Sciences, Shiraz, Iran; d Department of Family Medicine, School of Medicine, Shiraz University of Medical Sciences, Shiraz, Iran; e UTE University, General Directorate of Postgraduate Studies, 170527, Quito, Ecuador; f Department of Biomaterials, Saveetha Dental College and Hospital, Saveetha Institute of Medical and Technical Sciences, Chennai 600077, India; g Institute of Convergence Science, Korea University, Seoul 02841, Republic of Korea; h Department of Medical Nanotechnology, School of Advanced Medical Sciences and Technologies, Shiraz University of Medical Sciences, Shiraz, Iran

**Keywords:** QT interval prolongation, Lopinavir-ritonavir, COVID-19

## Abstract

A historical cohort study in southern Iran investigated the effect of lopinavir-ritonavir treatment, with or without azithromycin and/or hydroxychloroquine, on QT interval prolongation in 500 confirmed COVID-19 patients. The outcomes showed a notable increase in the mean corrected QT interval post-treatment, specifically from 415.75±46.848 ms to 437.91±50.455 ms. QTc prolongation was observed only in patients receiving lopinavir–ritonavir monotherapy who also presented with cardiovascular conditions, angina pectoris, or hypomagnesemia, whereas those receiving concomitant therapy with azithromycin or hydroxychloroquine did not exhibit this effect. Other than one-third of patients with comorbidities or additional QT-prolonging drugs, aside from hypertension and obesity, had clinically the same potential of QT prolongation, and only 10.6% of patients had the problem. The work stresses the significance of careful monitoring of cardiac health, especially for those patients who have electrolyte imbalances and cardiovascular diseases as underlying diseases when they use lopinavir-ritonavir with other QT-prolonging medications.

## Highlights

• QT interval increased significantly after Lopinavir-Ritonavir treatment in COVID-19 patients.
• QT prolongation mainly occurred in patients with heart disease, hypomagnesemia, or comorbidities.
• Only 10.6% of patients experienced clinically significant QT prolongation.
• Careful cardiac monitoring is vital for patients with electrolyte imbalances or cardiovascular issues.

## Graphical abstract

**Figure:**
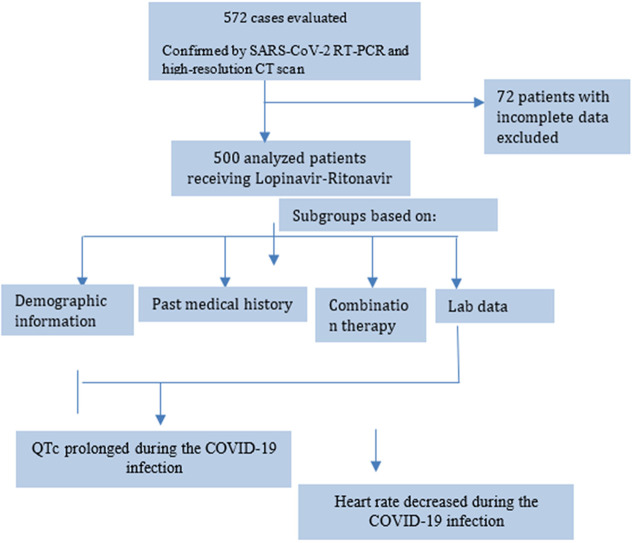

## 1 Introduction

The coronavirus disease 2019 (COVID-19) pandemic appears to have an abundance of countermeasures against it, despite having already passed. On the one hand, new strains of the severe acute respiratory syndrome coronavirus 2 (SARS-CoV-2) are emerging, and on the other, vaccines are being distributed. SARS-CoV-2 is a single-stranded positive-sense RNA virus that is a member of the Coronaviridae family (order Nidovirales), specifically within the Betacoronavirus genus. A high rate of mutation linked to genomic diversity and the emergence of new viral lineages is a characteristic of RNA viruses (Priyanka et al., 2021). Furthermore, studies on human coronaviruses and early data on SARS-CoV-2 indicate that both humoral and cellular adaptive immunity contribute to protective immune responses, highlighting the importance of vaccines eliciting robust activation of both arms of immunity. Furthermore, while decreasing severity in subsequent infections reflects priming of adaptive immunity, verified COVID-19 reinfection should not be discouraging. In practice, vaccination rather than spontaneous infection can produce herd immunity to SARS-CoV-2 (Priyanka et al., 2021). Given that mRNA can potentially be delivered via various platforms, significant progress has been made in the field of delivery, including advances in strategies for packaging mRNA and targeting its transport to specific organs in both clinical and experimental settings. As the stability, delivery, and related side effects of these vaccines are being monitored by the international scientific community, it is imperative to develop and modify effective drug and vaccine candidates against the most common infectious diseases to keep up with the emergence of novel infectious agents (Priyanka et al., 2023; Priyanka et al., 2023).

A number of effective vaccines have been developed more quickly as a result of scientific and technical advancements facilitated by international collaboration during the COVID-19 pandemic (Chen et al., 2023). Therefore, the ultimate success of the vaccination program depends on increased production, an effective logistics and distribution framework, an increase in implementation parameters such as efficiency and coverage, adherence to non-pharmaceutical mitigation measures, and the monitoring of in-field effectiveness, negative reactions, and vaccine failure cases, which would guide future vaccine and booster protocol adaptations (Choudhary and Singh, 2021; Choudhary et al., 2024). Thus, exploring new insights and alternatives in managing COVID-19 patients becomes an urgent need.

The first and only coformulated HIV-1 protease inhibitor, lopinavir/ritonavir, decreases the risk of opportunistic infections and malignancies (Cvetkovic and Goa, 2003). The therapeutic effectiveness of lopinavir/ritonavir has been shown in large clinical studies in patients who have never taken antiretrovirals before, as well as those who have. Treatment with this drug has demonstrated virologic and immunologic improvements in children, adolescents, and adults with HIV. The use of lopinavir/ritonavir monotherapy as a treatment option for some individuals is supported by smaller trials (Chandwani and Shuter, 2008). Despite its lifesaving effects against HIV, it shows detrimental effects on the cardiovascular system through inhibition of cardiac potassium channels like hERG, which can cause prolonged QT intervals that can lead to fatal arrhythmias like torsades de pointes (Ridjab et al., 2021). Furthermore, it is well recognized that COVID-19 itself can have an impact on the cardiovascular system and that viral infections put patients at risk for cardiac arrhythmias by causing metabolic problems, myocardial inflammation, and sympathetic nervous system activation (Istampoulouoglou et al., 2021). Furthermore, as this agent is an irreversible inhibitor of cytochrome CYP3A4, it is associated with multiple drug–drug interactions that should be carefully considered (Kandel and Lampe, 2021).

In the era of the COVID-19 pandemic, this agent was used in the short term against this disease due to its *in vitro* effects against SARS-CoV-2; however, no additional benefit was found for this combination in such patients (Kang et al., 2020; Patel et al., 2021). Medical professionals worldwide are actively pursuing pharmacological agents that can treat the disease and prevent its progression and related mortality. Existing pharmaceutical compounds are repurposed for the possible treatment of COVID-19 and examined in clinical trials to expedite the search for new therapy alternatives. The high number of patients receiving lopinavir-ritonavir in that period made it easier to assess the adverse effects of this agent and the effects of drug-drug interactions, specifically due to concomitant use of various agents prolonging the QT interval, including azithromycin, hydroxychloroquine, and chloroquine. This circumstance is crucial to comprehending the therapeutic safety of these drugs, especially when treating COVID-19, where their combination was investigated (Miatmoko et al., 2023).

Previous risk factors for QT interval prolongation in lopinavir-ritonavir use were advanced age, female sex, hypokalemia, acute myocardial infarction, concurrent use of two or more drugs with a prolongation effect on the QT interval, sepsis, and heart failure (HF)[16]. Three occurrences of significant corrected QT interval (QTc) prolongation events in patients with severe COVID-19 who were registered at a pharmacovigilance center and who were treated with lopinavir/ritonavir and hydroxychloroquine were reported by (Istampoulouoglou et al., 2021). These medications could have increased the risk of cardiovascular problems in three patients with severe COVID-19, according to the analysis of individual risk variables. These repurposed medications no longer have a place in treatment since they haven't demonstrated any improvement in COVID-19 outcomes since then. Furthermore, Zimmermanns et al. provided an overview of the cardiovascular side effects that were noted in three patients while they were receiving therapy for COVID-19 and discussed how they were related to hydroxychloroquine and lopinavir/ritonavir (Istampoulouoglou et al., 2021). Although this medication is no longer used in the context of COVID-19, it is still a valuable asset in the treatment of HIV infection. Thus, as an alternative exploration, in this study, the effect of lopinavir-ritonavir alone and in combination with other medications on the QT interval of patients admitted with a diagnosis of COVID-19 was assessed to increase the knowledge of physicians about the adverse effects of this drug in other contexts, like the treatment of patients with HIV/AIDS.

## 2 Methods

### 2.1 Patients

The current study followed a historical cohort protocol conducted on patients admitted with a diagnosis of COVID-19 in two referral centers of COVID-19 in Southern Iran. The study was performed from March 2021 to March 2022.

The inclusion criteria of the study were all patients above 14 years of age with a definitive diagnosis of COVID-19 pneumonia confirmed by SARS-CoV-2 RT-PCR and high-resolution CT scan, who were undergoing treatment with lopinavir-ritonavir alone or in combination with azithromycin and/or hydroxychloroquine, and who had at least one electrocardiogram before initiation of therapy. The exclusion criteria of the study were the unavailability of clinical data or insufficient data.

Based on a similar study by Chorin et al., considering a type I error of 0.05 and a study power of 80%, using Medcalc software version 19.6, the minimum sample size for the study to be statistically reliable was 491 (Chorin et al., 2020). Thus, 500 patients (197 patients from hospital A and 303 patients from hospital B) were enrolled in the study using a simple random sampling method.

Patient records were retrieved from the archive, and their baseline laboratory data at the time of admission and electrocardiograms were subsequently evaluated. HF was defined as a left ventricular ejection fraction of <40% or diastolic dysfunction of grade II or higher. Hypocalcemia was defined as a serum calcium level <8.8 mg/dL, hypokalemia as a serum potassium level of <3.5 mEq/L, hypomagnesemia as a serum magnesium level of <1.7 mg/dL, and hypoglycemia as a serum glucose level of <70 mg/dL. Blood counts were made using a Sysmex device, electrolyte analysis was conducted using the EasyLyte® device and its serologic kits. Other biochemical evaluations were made using the Dirui CS-400 device and Deltadarman (Tehran, Iran) serologic kits.

All patients were evaluated with a daily ECG. Then the QT interval of the patients was calculated by counting the small squares from the initiation of the Q wave to the end of the T wave, multiplying by 0.04 s at a paper speed of 25 mm/s. Corrected QT interval was measured using Bazett's Formula by dividing the QT interval by the square root of the RR interval. In cases of heart rate variability and different QTc measurements in various leads, the mean QTc in a 12-lead ECG was calculated for each patient by two researchers and verified in later stages of the study. Significant QT interval prolongation was defined as a QT interval longer than 500 ms, a QTc increasing of at least 60 ms after treatment, or pre-defined cutoff points for QTc for male and female patients (470 ms and 480 ms, respectively). The main ECGs addressed in the study were the baseline ECG before starting treatment and the ECG on the last day of admission.

### 2.2 Statistical analysis

All data were entered into the Statistical Package for Social Sciences version 19.0 (SPSS Inc., Chicago, IL, USA) for Windows for statistical analyses. The quantitative variables were reported as mean ± SD, and qualitative variables were reported as frequency and percentage. The patients were divided into multiple subgroups, and the groups were compared two-by-two using an independent two-sample T-test or Mann-Whitney U-test as appropriate for quantitative variables and a chi-squared test for qualitative variables. Pre- and post-treatment values in each group were measured using a paired T test or Wilcoxon Signed Rank test as appropriate. Two-sided p-values <0.05 and confidence intervals (CI) of 95% were considered to be statistically significant.

### 2.3 Ethical clearance

The current study was approved and financially supported by Shiraz University of Medical Sciences and was conducted in accordance with the Declaration of Helsinki. The study was approved by the Vice-Chancellor for Research and Technology and the local Ethics Committee of Shiraz University of Medical Sciences under the code IR.SUMS.MED.REC.1399.622. To consider ethical issues, the collected data were not revealed to anyone and remained confidential, except for the researchers; hence, patients' names were kept confidential.

## 3 Results

**Fig. 1:**
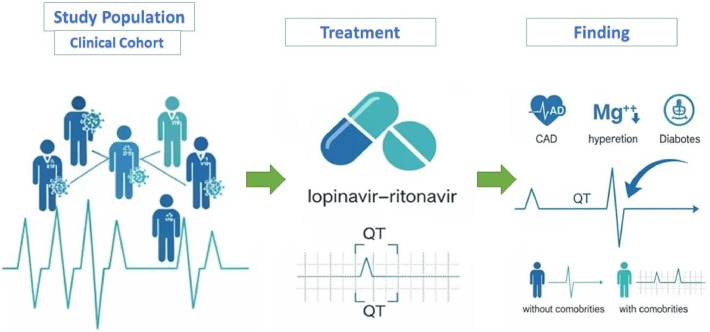
Flow diagram of the study.

**Fig. 2:**
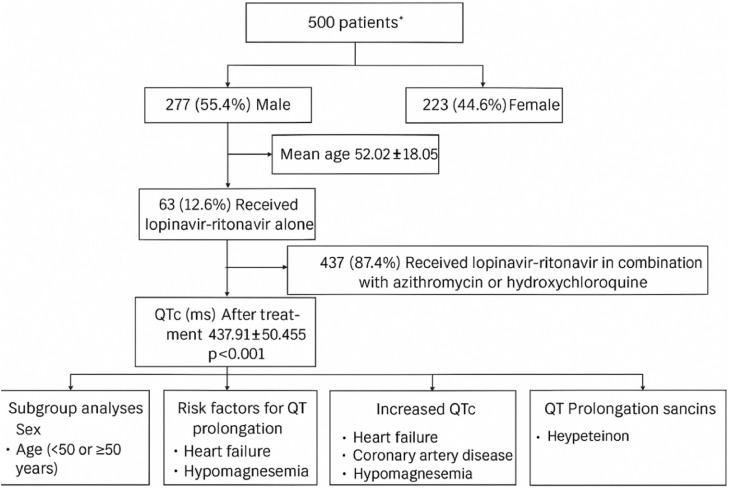
STROBE diagram of the investigation.

A total of 572 executive patients were evaluated, of whom 72 patients had insufficient data; thus, 500 patients were included in the study, of whom 277 patients (55.4%) were male, and 223 (44.6%) were female. The mean age of the patients was 52.02±18.05 years. In the study population, 63 patients (12.6%) received lopinavir-ritonavir alone, and the others received lopinavir-ritonavir in combination with azithromycin or hydroxychloroquine. Fig. 1 displays the study flow diagram, detailing exclusions, screening, and the derivation of the final study results. Fig. 2 indicates the STROBE diagram. Table 1 illustrates a high-risk cardiac population, with substantial electrolyte disturbances and notable mortality, emphasizing the importance of electrolyte correction and close ECG monitoring when prescribing QT-prolonging therapies.

**Table 1:** Baseline characteristics of the study population.

Variable		Mean/N	SD/percentage
**Sex**	**Male**	277	55.4%
	**Female**	223	44.6%
**Comorbidity**	**Diabetes Mellitus**	122	24.4%
	**Coronary Artery Disease**	102	20.4%
	**Hypertension**	81	16.2%
	**Renal insufficiency**	17	3.4%
	**Heart failure**	5	1.0%
**Outcome**	**Discharged**	447	89.4%
	**Deceased**	53	10.6%
**Hypokalemia**		33	6.6%
**Hypocalcemia**		210	42.0%
**Hypomagnesemia**		13	2.6%
**hypoglycemia**		16	3.2%
**Serum potassium level (mEq/L)**		4.34	0.61
**Serum Calcium level (mg/dL)**		8.82	0.68
**Serum Magnesium level (mg/dL)**		2.33	0.58
**Serum Glucose level (mg/dL)**		121.04	63.8

Based on demographic characteristics shown in Table 1, the cohort consisted of 500 patients, with 277 males (55.4%) and 223 females (44.6%). This shows a male predominance, which may reflect either the underlying epidemiology of patients receiving lopinavir–ritonavir or sex-specific differences in disease severity and treatment selection. Since sex hormones and ion channel regulation influence repolarization, the slight male predominance, patients' sex should be considered for risk stratification of QT prolongation; though the study later reported no significant QTc differences between sexes.

Regarding comorbidities, the most prevalent comorbidity was diabetes mellitus (DM) (24.4%), followed by coronary artery disease (CAD) (20.4%) and hypertension (16.2%). Renal insufficiency (3.4%) and HF (1.0%) were less common but clinically significant, as both conditions are known to amplify the risk of drug-induced QT prolongation. The relatively high prevalence of cardiometabolic comorbidities (diabetes, CAD, hypertension) suggests that this was a high-risk population for adverse cardiac events, given the interplay between these conditions and QT prolongation.

The majority of patients were discharged (447; 89.4%), while 53 patients (10.6%) Deceased during the study period. The mortality rate of ∼11% is clinically significant, raising questions about whether prolonged QTc, comorbidities, or electrolyte abnormalities contributed to adverse outcomes. A stratified outcome analysis by comorbidity or electrolyte status would clarify which factors most strongly predicted mortality.

Electrolyte disturbances are critical in modulating cardiac repolarization and arrhythmia risk. Although relatively uncommon, even mild hypokalemia (6.6%) can potentiate drug-induced QT prolongation. Strikingly common in this cohort. Hypocalcemia (42.0%) delays phase 2 of the cardiac action potential and predisposes to QT prolongation, making it a major risk factor in this population. While less frequent, hypomagnesemia (2.6%) is particularly dangerous, as it can precipitate torsades de pointes in the presence of QT-prolonging drugs. Less directly related to QT prolongation but important, as hypoglycemia (3.2%) can increase sympathetic activity and arrhythmogenic potential. The high prevalence of hypocalcemia (42%) stands out as the most clinically meaningful finding, indicating a potentially under-recognized contributor to QT prolongation in patients on antiviral therapy.

Mean 4.34 ± 0.61 mEq/L, within the normal range (3.5–5.0), but the standard deviation suggests that a subset of patients fell below the safe threshold, consistent with the 6.6% hypokalemia rate. Mean 8.82 ± 0.68 mg/dL, close to the lower end of normal (8.5–10.2), with many patients dipping into hypocalcemia, consistent with the very high 42% prevalence. Mean 2.33 ± 0.58 mg/dL, within normal limits (1.7–2.2), but the SD indicates variability, explaining the subset with hypomagnesemia. Mean 121.04 ± 63.8 mg/dL, suggesting a mixed population of diabetics and non-diabetics, with some experiencing hypoglycemia (3.2%). The wide SD (63.8) reflects significant variability, likely influenced by diabetes status and medication use.

QT prolongation risk was amplified not only by the drugs used (lopinavir–ritonavir, hydroxychloroquine, azithromycin in some cases) but also by baseline comorbidities (CAD, HTN, diabetes) and electrolyte abnormalities (notably hypocalcemia and hypokalemia).

In total, the mean corrected QT of the patients increased from 415.75±46.848 ms to 437.91±50.455 ms after treatment, which was statistically significant. Furthermore, patients showed decreased heart rate after treatment. Table 2 shows the changes in heart rate and QTc of the patients treated with lopinavir-ritonavir.

**Table 2:** Changes in heart rate and QTc of the patients treated with lopinavir-ritonavir.

Variable	Before treatment	After treatment	P-value
**QTc**	415.75±46.848	437.91±50.455	<0.001
**Heart rate**	93.00±18.976	83.45±16.248	<0.001

Overall, using lopinavir–ritonavir was linked to a statistically significant prolongation of the QTc. The mean QTc of the study group increased from 415.75 ± 46.848 ms at baseline to 437.91 ± 50.455 ms post-treatment, indicating a significant improvement in ventricular repolarization length (P < 0.05). This finding highlights the potential arrhythmogenic risk of the therapeutic regimen, as even modest QTc prolongation can predispose susceptible individuals to life-threatening ventricular arrhythmias, such as torsades de pointes.

After treatment, a simultaneous reduction in heart rate was seen alongside modifications in QTc. The drop in heart rate might be caused by autonomic control, physiological responses to medication, or the direct effects of lopinavir–ritonavir on the pathways that carry electrical signals through the heart. This decreasement may have significantly influenced QTc dynamics, as slower heart rates, when normalized for cycle time, can expose or exacerbate QT prolongation. In patients using protease inhibitors, the correlation between heart rate and QTc underscores the importance of comprehensive cardiac monitoring, particularly when used in conjunction with other drugs recognized to extend repolarization.

A detailed summary of the quantitative changes in heart rate and QTc observed in the cohort is provided in Table 2, which outlines pre- and post-treatment values, highlighting the magnitude of these electrophysiological alterations. This indicates that the treatment with lopinavir-ritonavir not only led to an increase in the QTc but also contributed to a reduction in heart rate among the patients. It suggests that while the immediate effects of the treatment are noteworthy, understanding the long-term consequences on cardiac health is crucial. By exploring the mechanisms behind these changes, researchers can better evaluate the overall safety and efficacy of the treatment for patients. Future studies should examine the underlying mechanisms responsible for these changes and assess their potential clinical significance.

The study population was stratified according to the presence of proposed risk factors for QT interval prolongation, and the impact of these factors on treatment outcomes was systematically assessed. In the subgroup of patients who received lopinavir–ritonavir monotherapy, the mean QTc following treatment was 423.40 ± 35.860 ms. By contrast, in patients who were administered lopinavir–ritonavir in combination with either azithromycin or hydroxychloroquine, the mean QTc increased to 440.01 ± 51.921 ms, representing a statistically significant prolongation (P = 0.004).

When stratified by sex and age, no significant differences were observed. Male and female patients exhibited comparable QTc values after treatment (P = 0.154), and similarly, patients younger than 50 years did not differ significantly from those older than 50 years (P = 0.476). Patients with DM or renal insufficiency had QTc intervals that were not statistically different from those of non-diabetic or renally intact patients, suggesting that the presence of metabolic and renal comorbidities did not appear to have a major impact. But hypertension turned out to be a clinically significant risk.

However, hypertension emerged as a clinically relevant factor. Patients with a history of hypertension exhibited a mean QTc of 435.86 ± 57.331 ms, compared with 438.31 ± 49.081 ms in normotensive individuals, a difference that reached statistical significance (P = 0.049). Furthermore, patients with structural or ischemic heart disease, including those with HF and coronary artery disease, demonstrated more pronounced QTc prolongation after treatment. Also, an electrolyte imbalance, especially low magnesium levels at admission, was linked to a greater likelihood of QTc prolongation. This suggests that baseline electrolyte problems may make certain treatment plans more likely to cause cardiotoxic problems.

These findings collectively emphasize that demographic factors, including sex and age, did not significantly affect QTc outcomes. However, cardiovascular comorbidities (such as hypertension, HF, and coronary artery disease) and electrolyte disturbances (specifically hypomagnesemia) are pivotal determinants of QT prolongation in patients receiving lopinavir–ritonavir-based therapies, especially when used in conjunction with QT-prolonging agents like azithromycin or hydroxychloroquine.

These data suggest that underlying cardiovascular problems and electrolyte imbalances may exacerbate the prolongation of QTc intervals in persons undergoing treatment. The subgroup analysis revealed variations in QTc response, as summarized in Table 3.

**Table 3:** Subgroup comparison of the effect of Lopinavir-Ritonavir on QT_c_.

Grouping variable		Before treatment	P-value	After treatment	P-value
**Interaction**	Lopinavir/Ritonavir alone	423.21±28.176	0.136	423.40±35.860	0.004
	In combination with Azithromycin or HCQ	414.67±48.886		440.01±51.921	
**Age**	<50 years old	407.77±38.425	<0.001	438.15±48.808	0.476
	≥50 years old	422.54±52.092		437.71±51.906	
**Sex**	Male	416.04±47.291	0.829	436.43±46.569	0.154
	Female	415.39±46.396		439.76±54.952	
**Diabetes**	No	417.84±42.988	0.023	435.58±51.551	0.068
	Yes	409.27±56.899		445.16±46.351	
**Renal insufficiency**	No	415.93±47.592	0.653	437.63±50.621	0.276
	Yes	410.47±13.593		446.00±46.132	
**Heart failure**	No	415.40±46.957	0.035	437.34±50.384	0.002
	Yes	450.00±0.000		494.60±2.408	
**Hypertension**	No	411.51±42.901	<0.001	438.31±49.081	0.049
	Yes	437.65±59.107		435.86±57.331	
**CAD**	No	412.96±44.962	0.001	435.05±52.021	0.001
	Yes	426.62±52.415		449.08±42.212	
**Hypokalemia**	No	416.00±47.408	0.757	438.21±51.300	0.516
	Yes	412.21±38.480		433.67±36.789	
**hypocalcemia**	No	408.78±42.983	0.001	436.86±46.683	0.392
	Yes	425.37±50.250		439.37±55.325	
**Hypomagnesemia**	No	415.76±47.294	0.655	437.41±51.000	0.031
	Yes	415.23±26.058		456.62±11.273	
**hypoglycemia**	No	416.38±47.349	0.057	438.65±51.012	0.015
	Yes	396.75±20.632		415.56±18.554	
**Outcome**	Discharged	414.50±45.843	0.132	438.55±48.702	0.412
	Deceased	426.28±53.938		432.53±63.657	

In the study population, a total of 53 patients (10.6%, 95% CI: 8.0%–13.6%) experienced QT interval prolongation during treatment. This occurrence highlights the clinically significant risk of ventricular repolarization anomalies in lopinavir–ritonavir-treated patients, especially when concomitant diseases or other QT-prolonging medications are present.

When stratified by comorbidity, the prevalence of clinically significant QT prolongation varied considerably across subgroups. Among patients with diabetes mellitus (DM), 15.6% exhibited QT prolongation compared to their non-diabetic counterparts, indicating a moderate yet significant risk. Conversely, CAD was associated with a markedly higher prevalence, with 32.4% of patients exhibiting QT prolongation. Chronic kidney disease (CKD) patients exhibited a prevalence of 29.4%, indicating the influence of renal dysfunction on medication elimination, electrolyte balance, and cardiac electrophysiology. The connection was considerably stronger in people with HF, where 60.0% of them exhibited QT prolongation. This shows that HF is one of the best clinical signs of arrhythmic risk. Patients with hypomagnesemia at admission had a significantly elevated risk of QT prolongation (46.2%) in contrast to those with normal magnesium levels.

Binary logistic regression analysis was conducted to more precisely quantify the relative risk associated with these characteristics. This research showed that HF (OR = 13.350) posed the highest risk of QT prolongation, indicating that individuals with structural heart disease are especially susceptible to the arrhythmogenic effects of antiviral treatments. CAD (OR = 9.039) and concomitant medication with azithromycin and/or hydroxychloroquine (OR = 8.374) were identified as significant independent predictors, aligning with the cumulative QT-prolonging effects of polypharmacy and ischemic myocardial substrate irregularities. Hypomagnesemia (OR = 8.024) also appeared as a significant factor, validating the role of electrolyte balance in influencing cardiac repolarization. CKD (OR = 3.776) was associated with a moderate but significant increase in risk, likely due to impaired renal clearance of drugs and electrolytes. Finally, DM (OR = 1.886) was associated with nearly a two-fold increase in the odds of QT prolongation, indicating a contributory yet less pronounced effect relative to cardiovascular comorbidities and electrolyte imbalances.

A detailed overview of these associations, including odds ratios, is presented in Table 4, which delineates the relative contributions of comorbidities, concomitant therapies, and electrolyte abnormalities to the development of QT prolongation in this patient population.

**Table 4:** Evaluation of the risk factors of QT prolongation.

Variable		Chi-squared test		Binary logistic regression	
		Prevalence	P-value	OR	P-value
**Sex**	Male (N = 277)	26 (9.4%)	0.326	reference	Reference
	Female (N = 223)	27 (12.1%)		1.330	0.327
**Interaction**	No (N = 63)	1 (1.6%)	0.008	reference	reference
	Yes (N = 437)	52 (11.9%)		8.374	0.037
**Diabetes**	No (N = 378)	34 (9.0%)	0.040	reference	reference
	Yes (N = 122)	19 (15.6%)		1.866	0.043
**CAD**	No (N = 398)	20 (5.0%)	<0.001	reference	reference
	Yes (N = 102)	33 (32.4%)		9.039	<0.001
**HTN**	No (N = 419)	43 (10.3%)	0.577	reference	reference
	Yes (N = 81)	10 (12.3%)		1.232	0.578
**CKD**	No (N = 483)	48 (9.9%)	0.025	reference	reference
	Yes (N = 17)	5 (29.4%)		3.776	0.016
**HF**	No (N = 495)	50 (10.1%)	<0.001	reference	reference
	Yes (N = 5)	3 (60.0%)		13.350	0.005
**hypokalemia**	No (N = 467)	49 (10.5%)	0.768	reference	reference
	Yes (N = 33)	4 (12.1%)		1.177	0.769
**hypocalcemia**	No (N = 290)	27 (9.3%)	0.271	reference	reference
	Yes (N = 210)	26 (12.4%)		1.376	0.272
**hypomagnesemia**	No (N = 487)	47 (9.7%)	<0.001	reference	reference
	Yes (N = 13)	6 (46.2%)		8.024	<0.001
**hypoglycemia**	No (N = 484)	51 (10.5%)	0.683	reference	reference
	Yes (N = 16)	2 (12.5%)		1.213	0.802

## 4 Discussion

In this historical cohort study involving 500 hospitalized patients with COVID-19, we evaluated the effects of lopinavir–ritonavir alone and in combination with azithromycin or hydroxychloroquine on cardiac repolarization. Our findings demonstrated a significant increase in the overall mean QTc interval by 22.16 ms following treatment, confirming that antiviral therapy in this context exerts measurable effects on ventricular repolarization. Importantly, the magnitude of QT prolongation differed between treatment groups: patients receiving lopinavir–ritonavir alone showed negligible changes in QTc (0.19 ms), whereas those treated with lopinavir–ritonavir in conjunction with azithromycin and/or hydroxychloroquine exhibited a much larger increase (25.33 ms). This highlights the synergistic effect of combining multiple QT-prolonging drugs, consistent with prior reports of increased arrhythmic risk with polypharmacy in COVID-19.

When analyzing patient subgroups irrespective of drug exposure, certain comorbidities and laboratory findings were associated with longer QTc intervals. Patients with HF, CAD, and hypomagnesemia consistently demonstrated more pronounced QT prolongation. These results align with mechanistic understanding, as structural heart disease alters myocardial repolarization reserve, while magnesium deficiency disrupts ion channel function, predisposing to arrhythmia. Conversely, patients with hypertension and hypoglycemia exhibited shorter QTc intervals in our cohort. The latter finding is intriguing because prior studies have reported QT prolongation during hypoglycemia, likely due to catecholamine surge and delayed repolarization. The contradictory result in our study may reflect measurement variability, concomitant drug exposures, or compensatory autonomic influences and requires further investigation in larger, mechanistic studies.

When analyzed together, these data demonstrate a clear risk hierarchy. The biggest risks come from structural heart disease (HF, CAD), polypharmacy (lopinavir–ritonavir with azithromycin or hydroxychloroquine), and electrolyte problems (hypomagnesemia). Renal insufficiency and diabetes also contribute, albeit to a lesser extent. The findings indicate that patients with these conditions require individualized regimens for cardiac monitoring. To reduce the risk of arrhythmia, it is essential to assess and correct electrolyte imbalances, particularly hypomagnesemia and hypokalemia, prior to therapy, and to exercise caution when co-administering QT-prolonging medications.

Our results corroborate earlier observations in the literature. A study reported that nearly 30% of patients treated with hydroxychloroquine and azithromycin developed QTc prolongation greater than 40 ms, and 11% reached severely prolonged QTc intervals (Ridjab et al., 2021). This was in line with our results since patients receiving azithromycin and/or hydroxychloroquine with lopinavir-ritonavir showed significant prolongation in their QTc, which was not the case in patients not receiving those two medications.

In a report by the French network of pharmacovigilance centers, cardiac adverse effects of lopinavir-ritonavir, hydroxychloroquine, chloroquine, and azithromycin were assessed. The authors found that 86% of the reactions were attributable to hydroxychloroquine alone or with azithromycin. Lopinavir-Ritonavir was used in only 14% of the cases, none of which were clinically significant (Gérard et al., 2020). This was in line with our results showing that patients with adjunctive treatment of hydroxychloroquine and lopinavir-ritonavir had significantly higher QTc after treatment, and the effects of those agents are much stronger than those of lopinavir-ritonavir. These findings are consistent with our data, where lopinavir–ritonavir monotherapy was associated with minimal QTc changes in our cohort, although definitive safety cannot be established; concomitant therapy with macrolides or hydroxychloroquine significantly increased QTc prolongation.

In a study by Sarapa et al., in healthy patients, the changes in the QT interval after administration of ritonavir and moxifloxacin versus moxifloxacin and placebo were evaluated. They concluded that 100 mg did not show a QT interval prolongation effect in patients (Sarapa et al., 2008). Our data extend this observation to a larger, real-world population of COVID-19 patients, where lopinavir–ritonavir alone did not significantly prolong QTc, suggesting its arrhythmogenicity may be limited in short-term use. However, when combined with azithromycin, the pharmacokinetic interaction of lopinavir as a cytochrome P450 3A4 inhibitor can elevate serum concentrations of azithromycin, thereby enhancing the QT-prolonging effect. This potential for synergistic cardiotoxicity underscores the need for careful co-prescription.

In a study by Cao in COVID-19 patients, the addition of lopinavir to the standard of care was assessed. They found that the addition of lopinavir to the standard of care increased the risk of QT prolongation (Cao et al., 2020). This finding might be misinterpreted since "standard of care" includes appropriate use of antibiotics in patients with superimposition of bacterial pneumonia, which would normally include a macrolide and a third-generation cephalosporin or a fluoroquinolone unless other conditions, such as hospital-acquired pneumonia or ventilator-associated pneumonia, are suspected, which would prompt stronger antibiotic regimens (Martin-Loeches et al., 2023). In that study, 95.0% of the patients had received antibiotic therapy. Thus, macrolides like azithromycin would have played an important role in the QT prolongation of the patients in that study. With the fact that lopinavir, as a cytochrome P450 3A4 inhibitor, increases the serum concentration and adverse events of azithromycin, the synergistic effects of these agents in QT prolongation should be kept in mind (Yeh et al., 2006).

In another study, QT interval prolongation was accounted for as an adverse effect of lopinavir-ritonavir, hydroxychloroquine, chloroquine, and azithromycin. The authors found that age older than 68 years, female sex, hypokalemia, QT interval longer than 450 ms at time of admission, acute myocardial infarction, using two or more QT-prolonging agents, sepsis, and HF increased the risk of this condition (Mercuro et al., 2020).

In our study, patients with hypomagnesemia had longer QTc, which might be a confounding factor since hypomagnesemia itself has a QT prolongation effect (Yang et al., 2020). Patients with hypoglycemia at admission had shorter QT, which contradicts previous research; thus, it needs further investigation (Beom et al., 2013). Patients with HF and coronary artery disease also showed more prolonged QT intervals in our study, which might be due to their underlying cardiologic condition, especially since those patients had more prolonged initial QTc intervals before treatment.

Lastly, although our study included a large sample size that enhances the generalizability of the findings, several limitations should be acknowledged. The timing of ECG measurements in relation to medication delivery was not standardized, potentially introducing heterogeneity in QTc evaluation. Furthermore, dynamic fluctuations in electrolyte and glucose levels during hospitalization were not monitored longitudinally, limiting our ability to fully characterize the temporal relationships between laboratory abnormalities and QTc prolongation. Notwithstanding these constraints, the investigation offers significant evidence about the comparative safety of lopinavir-ritonavir monotherapy against its administration with additional QT-prolonging medicines.

One of the main drawbacks of the study was the lack of consideration of the timing of drug administration with electrocardiographic measurements and the course of lab data alterations during hospitalization. But the power of the study lies in the large sample size. Despite the relatively large sample size of the patients evaluated in this study, the number of long QT events was relatively small, which results in overfitting of the regression models; furthermore, further studies are needed for verification of our results. However, since combination therapy with Lopinavir/Ritonavir and other QT-prolonging agents such as Azithromycin or Hydroxychloroquine imposes a life-threatening risk to the patients, extensive ethical consideration should be made for other studies. Due to such ethical considerations, a larger sample size collection was not possible in our centers since using the combination was ceased after evaluation of such cardiovascular complications for proper patient safety. Furthermore, the patients in our study received other drugs such as antibiotics (including Piperacillin-Tazobactam, Ampicillin-Sulbactam, Vancomycin, Meropenem, Ceftriaxone, Cefepime, Tobramycin, Colistin, and Levofloxacin among others), other Antihistamines such as Diphenhydramine, Cetirizine, Loratadine, and Desloratadine among others. Care was taken to exclude patients receiving other QT-prolonging agents, such as Diphenhydramine or Levofloxacin, to emphasize the specific interaction mentioned in this study.

In summary, our results demonstrate that lopinavir–ritonavir monotherapy was not associated with clinically significant QTc prolongation in our cohort, whereas its concomitant use with azithromycin or hydroxychloroquine markedly elevates the risk of QT prolongation, especially in patients with cardiovascular comorbidities and electrolyte imbalances. These results highlight the necessity of risk assessment, ECG monitoring, and prudent medication selection to mitigate the risk of potentially fatal arrhythmias in COVID-19 patients.

## 5 Conclusions

In this historical cohort study of 500 COVID-19 patients, we demonstrated that treatment with lopinavir–ritonavir was associated with a statistically significant overall increase in QTc interval, with a mean prolongation of 22.16 ms. Importantly, while lopinavir–ritonavir monotherapy was not associated with clinically meaningful QTc prolongation, the addition of azithromycin and/or hydroxychloroquine led to a pronounced increase in QTc, highlighting the synergistic arrhythmogenic potential of these drug combinations.

Our results align with previous research that identified hydroxychloroquine and azithromycin as significant factors in drug-induced QT prolongation, but lopinavir–ritonavir alone seems to present minimal risk, especially with short-term usage. Moreover, we found that individuals with HF, coronary artery disease, and hypomagnesemia were considerably more susceptible to QTc prolongation, consistent with previously identified high-risk cohorts. Interestingly, patients with hypoglycemia exhibited shorter QTc intervals, a result that diverges from earlier research and warrants further investigation.

The present study underscores the need for careful patient selection, baseline risk assessment, and vigilant ECG and electrolyte monitoring when prescribing QT-prolonging therapies in COVID-19, particularly in individuals with pre-existing cardiovascular disease or electrolyte imbalances. Although our relatively large sample size enhances the reliability of these findings, limitations include the absence of standardized timing for ECG recordings relative to drug administration and the lack of longitudinal follow-up of laboratory parameters throughout hospitalization.

According to our findings, lopinavir–ritonavir by itself appears to be relatively secure in the short term in terms of cardiac repolarization; however, when used in conjunction with azithromycin or hydroxychloroquine, the risk of QT prolongation is significantly increased. To reduce the danger of potentially fatal arrhythmias, clinicians should prioritize customized monitoring techniques and exercise caution when it comes to such combinations.

**Abbreviations**
**SARS-CoV-2**: Severe Acute Respiratory Syndrome Coronavirus 2.
**HTN**: Hypertension.
**HF**: Heart Failure.
**CKD**: Chronic Kidney Disease.
**CAD**: Coronary Artery Disease.
**DM**: Diabetes Mellitus.

## Funding

This study was financially supported by 10.13039/501100004320Shiraz University of Medical Sciences (Grant No. 21994).

## CRediT authorship contribution statement

**Ali Jangjou:** Conceptualization, Investigation, Visualization, Methodology, Writing – original draft. **Payman Izadpanah:** Conceptualization, Investigation, Visualization, Writing – original draft. **Mostafa Moqhadas:** Conceptualization, Data curation, Formal analysis, Writing – review & editing. **Hosein Faramarzi:** Conceptualization, Data curation, Visualization, Writing – review & editing. **Mohammad Hossein Fahimi:** Conceptualization, Validation, Visualization, Writing – review & editing. **Hamid Ghazipoor:** Conceptualization, Validation, Visualization, Writing – review & editing. **Hadid Hamrah:** Project administration, Supervision, Validation, Visualization, Writing – review & editing. **Hesam Kamyab:** Conceptualization, Software, Visualization, Writing – review & editing.

## Declaration of competing interest

The authors declare that they have no known competing financial interests or personal relationships that could have appeared to influence the work reported in this paper.

## Data Availability

Data will be made available on request.
